# The comparison of plasma zonulin levels between symptom exacerbation and treatment response periods in schizophrenia: a case-control study with follow-up

**DOI:** 10.1192/j.eurpsy.2023.1318

**Published:** 2023-07-19

**Authors:** O. Aydın, T. Kocabaş, A. Sarandöl, A. Muştucu, E. Onur, A. Esen-Danacı

**Affiliations:** 1 International University of Sarajevo, Sarajevo, Bosnia and Herzegovina; 2 Celal Bayar University, Manisa; 3 Uludağ University, Bursa, Türkiye

## Abstract

**Introduction:**

Plasma zonulin is acknowledged to be a biomarker for intestinal permeability. Previous studies have demonstrated significant relationships regarding potential effects of zonulin in several major psychiatric conditions including attention deficit and hyperactivity disorder, autism spectrum disorder and depression, however its role in schizophrenia still remains unclear.

**Objectives:**

We aimed to identify the alterations of plasma zonulin levels between the different periods of the illness and we strive to reveal the associations between plasma zonulin levels and symptoms in patients with chronic schizophrenia.

**Methods:**

30 patients with schizophrenia and 29 healthy controls participated in the study. Sociodemographic data form and Positive and Negative Syndrome Scale (PANSS) were administered. Blood sampling was performed for patients who are in exacerbation and following treatment response periods along with the healthy controls. ELISA method was utilized to measure plasma zonulin levels.

**Results:**

The groups did not differ in plasma zonulin level comparisons. Plasma zonulin did not fluctuate between the symptom exacerbation and treatment response periods of the patients. Besides, plasma zonulin level was found to be associated with passive/apathetic social withdrawal, active social avoidance, and somatic concern items of the PANSS in a negative direction.

**Conclusions:**

This is the first follow-up study in the literature that assesses plasma zonulin in patients with schizophrenia. The measurement of plasma zonulin may not be a convenient parameter in distinguishing symptom exacerbation and treatment response periods in chronic schizophrenia, nevertheless it may have implications on reduced social interaction and somatic symptoms. Our study can provide better insight for future studies to be more cautious while interpreting the associations of plasma zonulin levels with psychiatric disorders.

**Disclosure of Interest:**

None Declared

